# IgE-dependent human basophil responses are inversely associated with the sarcoplasmic reticulum Ca^2+^-ATPase (SERCA)

**DOI:** 10.3389/fimmu.2022.1052290

**Published:** 2023-01-06

**Authors:** Anette T. Hansen Selnø, Vadim V. Sumbayev, Bernhard F. Gibbs

**Affiliations:** ^1^ School of Pharmacy, University of Kent, Chatham Maritime, United Kingdom; ^2^ Department of Human Medicine, University of Oldenburg, Oldenburg, Germany

**Keywords:** basophils, SERCA, IgE receptor, histamine, inhibitory signaling

## Abstract

Basophils crucially contribute to allergies and other Th2-driven diseases by rapidly releasing inflammatory and immunomodulatory mediators following high-affinity IgE-receptor crosslinking. Although these basophil-mediated responses depend on sensitization with antigen-specific IgE, this does not necessarily predict clinical symptom severity. It is thought that the balance of early stimulatory (e.g. SYK) and inhibitory (e.g. SHIP-1) intracellular signals are associated with basophil responsiveness, which is also critically dependent on calcium mobilization. Previous studies suggest that the sarcoplasmic reticulum Ca^2+^-ATPase (SERCA2), which regulates cytosolic calcium levels, may be inversely associated with airway smooth muscle reactivity in asthma. Since basophils are implicated in asthma severity, our aims were to address whether SERCA2 is implicated in human basophil responses, especially following IgE-mediated activation. Human basophils were obtained from buffy coats, following research ethics approval, and further purified by immunomagnetic cell sorting. Expressions of SERCA2, and other isoforms, were determined by Western blotting in parallel to measuring IgE-dependent histamine releases from the same donors. The effects of a SERCA-activator and inhibitor were also assessed on their abilities to modulate basophil histamine release. We observed an inverse correlation between basophil responsiveness to IgE-dependent stimulation and SERCA2 expression. Thapsigargin, a highly-specific SERCA inhibitor, stimulated basophil histamine release and potentiated IgE-dependent secretion of the amine. Conversely, disulfiram, a SERCA activator, inhibited IgE-dependent basophil activation. The results obtained from this exploratory study indicate that SERCA2 may be an additional regulator of basophil reactivity alongside early excitatory or inhibitory signal transduction pathways.

## 1 Introduction

Basophils contribute to the severity of allergic reactions by their capacity to rapidly release histamine and leukotriene C4. They also play a pivotal role in initiating and sustaining Th2-type pro-allergic immune responses by releasing IL-4 and IL-13, alongside various inflammatory cytokines such as TNFα. Despite their relative scarcity, especially to mast cells with which they share the ability to release pro-inflammatory mediators by IgE-dependent mechanisms, animal models of allergy and immunity to parasites have demonstrated that basophils play a non-redundant role in initiating Th2 responses and in chronic allergic inflammation (reviewed in (1). These immunomodulatory attributes are thought to occur following the migration of circulating basophils to tissues affected by allergic inflammation as well as to associated lymphatic tissues. Mouse models have also shown that basophils may also differentially contribute to anaphylaxis (2–4). In humans, basophil activation tests and a rapid decline in circulating basophils have shown a strong correlation to the severity of severe allergic reactions to foods and insect venoms (5–7), further highlighting a role for this cell type in anaphylaxis.

The crosslinking of IgE molecules bound to high-affinity IgE receptors (FcεRI) by allergens crucially determines basophil activation in humans, whereas in mice IgG-mediated anaphylaxis due to basophil activation may also occur (3), underlining the importance for detailed studies of human basophil function. Interestingly, although the degree of human basophil sensitization with antigen-specific IgE is important for enabling allergen-induced cell activation, the severity of clinical symptoms of basophil-driven diseases crucially depends on the basophil phenotype, especially in terms of the concept of “releasability” (8). Previous studies have shown that the releasability of basophils, regarding their strength of activation to IgE-dependent triggers, is fundamentally regulated by various intracellular signals. These include the expression and phosphorylation of early stimulatory kinases, especially the spleen tyrosine kinase SYK (9), and the subsequent activation of downstream kinases such as phosphatidylinositol 3-kinase (PI 3-kinase) (10, 11) and p38 mitogen-activated kinases (p38 MAPK) (12). Basophil stimulation is, however, also downregulated by inhibitory intracellular signals, notably the Src homology 2 (SH2) domain containing inositol polyphosphate 5-phosphatase 1 (SHIP1), which reduces both basophil and mast cell function (13–17). SHIP-1 is also associated with the concept of basophil non-releasers (16), which is observed in up to 20% of donors at any given time where basophils are unable to respond to IgE-dependent stimuli, unrelated to the expression of IgE and FcεRI *per se* (18). This anergic state of human basophils may also be achieved by targeting various inhibitory receptors such as CD300a (19, 20), Allergin-1 (4), FcγRIIb (21) and siglec-7 (22). Basophil responsiveness (releasability) is therefore intricately governed by the balance of stimulatory and inhibitory signaling.

SYK and PI 3-kinase phosphorylation ultimately leads to the activation of phospholipase C and the subsequent synthesis of inositol trisphosphate (IP_3_). IP_3_ is crucially responsible for the increase in intracellular free calcium that drives the opening of calcium-sensitive calcium channels, allowing for the influx of extracellular calcium ions into basophils, without which no mediator release occurs. Calcium mobilization in basophils is therefore an essential step in determining basophil responses. IP_3_-mediated leakage of calcium ions from intracellular stores, such as the sarcoplasmic reticulum, is potentially offset by the sarcoplasmic reticulum Ca^2+^-ATPase (SERCA2). Interestingly, SERCA2 expressions in airway smooth muscle cells have been reported to be inversely associated with airway inflammation (23) and asthma severity (24). Moreover, it has long been known that Ca^2+^-ATPase blocker, thapsigargin, activates human basophils (25). These cells have been strongly implicated in asthma severity, particularly during allergic late-phase reactions, where their increased numbers within the lungs and their activation are associated with severe outcomes, including death (26, 27). It is notable that basophils from allergic asthma patients displayed substantially higher magnitudes of histamine release induced by thapsigargin than non-allergic controls (28), indicating that SERCA2 may play a role in the severity of symptoms in basophil-driven diseases. IgE-dependent basophil activation is known to be greatly enhanced by priming cytokines, such as IL-3, IL-5 and GM-CSF, which are elevated in allergic asthma (29–31). Remarkably, these cytokines, which by themselves are poor stimuli of basophil degranulation, can cause substantial release of histamine from basophils in the presence of sub-optimal concentrations of thapsigargin, further indicating that depleting intracellular Ca^2+^ stores critically activates human basophils receptor-mediated histamine release (32).

Given the association of SERCA2 in asthma and that basophils are implicated in asthma severity, our aims were to address the principle of whether SERCA2 governs human basophil responses, especially in relation to IgE-mediated signaling. Our proof-of-concept study suggests that basophil releasability to IgE-dependent activation is, at least in part, determined by SERCA2 expression and possibly by other SERCA isoforms.

## 2 Materials and methods

### 2.1 Isolation of human basophils

Basophils were obtained from buffy coats, following ethical approval from the National Health Service (NHS) Research Ethics Committee (reference number 07/Q1206/3), purchased from the NHS Blood and Transfusion service. Basophils were first isolated by Ficoll-density centrifugation (using Ficoll-Paque Plus, GE Healthcare, Uppsala, Sweden) and purified further by immunomagnetic cell selection (negative selection) using commercial isolation kits (EasySep Human Basophil Enrichment Kit, STEMCELL Technologies, Grenoble, France) as previously reported (33, 34). Basophil numbers and purities were verified by light microscopy using alcian blue staining and a Fuchs-Rosenthal haemocytometer. Mean basophil purity obtained following immunomagnetic selection was 91.6 ± 1.2%.

### 2.2 Western blot analysis

Aliquots of purified basophils (1-2 x 10^5^ cells) were pelleted by centrifugation, and then lysed by vigorous mixing with lysis buffer containing 50 mM Tris-HCl pH 7.5, 5 mM EDTA, 10mM EGTA, 5 mM DTT, 1% Nonidet P-40, 1mM PMSF, 100 ug/ml aprotonin, 20 ug/ml leupeptin and 10 mM benzamidine. An equal volume of 2x-concentrated Laemmli sample buffer was then added to the lysed basophils which were then heated to 99°C, with agitation, for 2 min before storage at -80°C. Proteins were separated by 12% sodium dodecyl sulfate–polyacrylamide gel electrophoresis (SDS-PAGE) and then blotted onto nitro-cellulose membranes. Prestained molecular weight rainbow markers (Bio-Rad Laboratories Ltd, Watford, UK) were also included for each SDS-PAGE run. Membranes were blocked for 4 h in 5% skimmed milk dissolved in TBST buffer (20 mM Tris-HCl, pH 7.5, 137 mM NaCl, 0.1% Tween 20) with gentle agitation. After 3 x 5 min washes in TBST, membranes were incubated overnight (at 4°C) with primary antibodies, directed against human SERCA2 (mouse monoclonal (ab2817) purchased from Abcam, Cambridge, UK). Membranes were then successively washed (4 x 5 min, TBST) followed by incubation with anti-mouse horse radish peroxidase-conjugated secondary antibodies for 2 h with gentle agitation. After washing, unbound secondary antibody proteins were visualized by autoradiography according to the manufacturer’s instructions (ECL plus, Amersham, Buckinghamshire, UK). After detection, membranes were stripped for 10-20 min using Re-blot plus reagent (Chemicon, Chandlers Ford, UK), washed in TBST (4 x 5 min) and reprobed. Beta-actin expressions were measured in order to validate equal protein loading using mouse monoclonal HRP-conjugated antibodies (ab20272). Densitometric analysis was performed using ImageJ and the relative band densities of SERCA were normalized to respective band densities of β-actin and adjusted to control samples.

### 2.3 Cell treatments

The IgE-dependent reactivity of basophils from donors used to detect SERCA expressions by Western blot was determined by assessing histamine release. Briefly, vials containing 50-100 x 10^4^ basophils, resuspended in HEPES-buffered Tyrode’s solution (buffer), were warmed for 15 min at 37°C before stimulation with 1 µg/ml goat anti-human IgE (ϵ-chain specific, Sigma-Aldrich, St. Louis, USA) for 30 min, alongside unstimulated controls. Reactions were then terminated by adding ice-cold calcium-free buffer and centrifuging vials for 2 min at 1000 x g. Histamine content in the supernatants and cell pellets, which were diluted as required and lysed with perchloric acid (4%), was assessed using a spectrofluorometric autoanalyzer, based on the method reported by Shore et al. (35). Percentage histamine releases were determined from the total histamine content in the sum of pellet and supernatant tubes.

In a separate series of experiments, the effects of disulfiram (a SERCA activator) on basophil histamine release were investigated by preincubating isolated basophils with various concentrations of disulfiram (together with buffer controls) for 15 min at 37°C before stimulation with anti-IgE and assessment of histamine releases as described above. Part of the remaining cell pellets were also subjected to MTS viability assays. Briefly, cells were incubated with 3-(4,5-dimethylthiazol-2-yl)-5-(3-carboxymethoxyphenyl)-2-(4-sulfophenyl)-2H-tetrazolium (MTS) and absorbance was measured at 490 nm using a plate reader, according to the manufacturer’s instructions (Promega UK Ltd., Southampton, UK).

### 2.4 Statistical analysis

Experiments were repeated using different basophil donors and results were first tested for normal distribution using the Shapiro-Wilk test. For normally-distributed data, a one-way ANOVA followed by Holm-Bonferroni correction was employed to assess statistically significant differences when multiple comparisons were made and a paired Student’s t-test when comparing two events. The potential association between IgE-dependent basophil reactivity and SERCA2 expression was analyzed using linear regression analysis as well as Spearman’s rank correlation- and Pearson correlation coefficients. Statistical probabilities (p) were expressed as *, where p < 0.05, **p < 0.01 and ***p < 0.001, unless shown otherwise.

## 3 Results

In agreement with previous reports (25, 28, 32), we first confirmed that thapsigargin activates human basophils and, at sub-optimal concentrations, potentiated IL-3-stimulated basophil histamine release (**Supplementary Figure 1**). IgE-mediated basophil activation was only weakly potentiated by thapsigargin, whereas mediator secretion induced by the bacterial peptide secretagogue, fMLP, was not at all enhanced. We could further verify that thapsigargin induced substantial calcium mobilization in basophils but, in contrast to the calcium ionophore A23187, the kinetics of calcium mobilization were slower in the presence of extracellular calcium (**Supplementary Figure 2**). Interestingly, in the absence of extracellular calcium in the buffer, thapsigargin had very similar effects (and at the same rate) compared to A23187, supporting its mode of action as an intracellular calcium liberator due to SERCA blockade.

**Figure 1:**
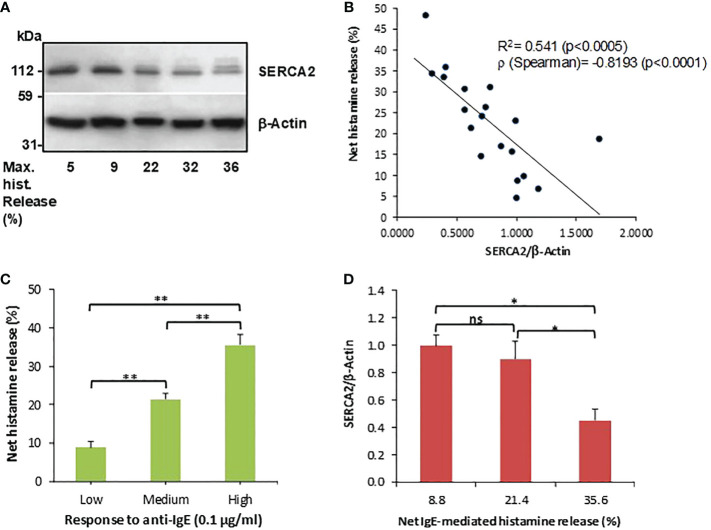
SERCA2 expressions and IgE-dependent histamine release from human basophils are negatively correlated. **(A)** SERCA2 expressions in unstimulated purified basophils, as determined by Western blotting, representative for a total of 19 separate basophil donors investigated. **(B)** Densitometric analysis of SERCA2 expressions plotted against the corresponding net anti-IgE-induced histamine release from the same basophil donors (n=19). **(C)** Histamine releasability to IgE-dependent stimulation grouped to low (<15% net release, n=5), medium (15 - 30% net release, n=8) and high (>30% net release, n=6) responders which were employed in **(D)** showing that SERCA2 expressions are clearly and significantly reduced in high responder basophils. Histamine data are shown as means ± SEM. * and ** indicate significant (p<0.05 and p<0.01, respectively) differences as determined by a one-way ANOVA followed by Holm-Bonferroni correction. ns, not significant.

Our preliminary investigations indicated that human basophils variably express SERCA2 and, to a lesser extent, also SERCA3, but we failed to observe SERCA1 expressions in these donors (**Supplementary Figure 3**). Because Mahn et al. (24) previously reported that SERCA2 is inversely associated with asthma severity, and given that basophils show relatively high SERCA2 expressions compared to other isoforms, we wished to more closely examine whether SERCA2 expressions in human basophils is associated with their releasabilty to IgE-dependent stimulation. Indeed, basophils isolated from healthy donor buffy coat blood differentially expressed SERCA2 (**Figure 1A**) and, overall, this expression appeared to be clearly inversely associated with IgE-dependent histamine release from the same donors (**Figure 1B**). The negative correlation between SERCA2 expression and corresponding IgE-dependent histamine release was highly statistically significant, despite several outliers (see also **Supplementary Table 1** for a summary of all the data shown in **Figure 1**). In contrast, spontaneous histamine secretion was not significantly associated with SERCA2 expression (R^2^ = 0.1; p>0.2). Because IgE-dependent basophil activation between different donors (even from healthy individuals) is highly variable, we grouped the donors into low, medium and high responders to anti-IgE stimulation. We observed that the expression of SERCA2 was diminished most noticeably in the high responder group (net histamine release >30%; **Figures 1C** **, D**), indicating that SERCA2 may, at least in part, be involved in governing low IgE responder phenotypes in addition to other known regulatory signals (especially SYK and SHIP-1).

**Figure 2:**
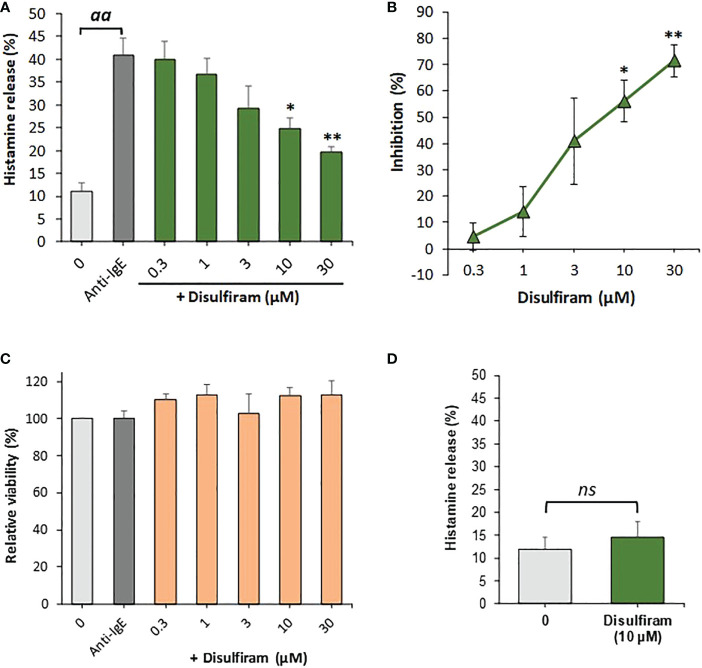
Disulfiram inhibits IgE-dependent histamine release from human basophils. **(A)** Basophils were preincubated with or without various concentrations of disulfiram for 15 min at 37°C before stimulation with anti-IgE (1µg/ml) for 30 min (n=5). Results are expressed as relative percentage histamine releases from which **(B)** the percentage inhibition of anti-IgE-induced histamine release was calculated from net histamine releases (corrected for spontaneous release controls). *aa* denotes significant (p<0.01) differences between anti-IgE stimulated positive controls from spontaneous release controls, * and ** indicate significant (p<0.05 and p<0.01, respectively) differences between anti-IgE alone and basophils incubated with disulfiram at the indicated concentrations as determined by a one-way ANOVA followed by Holm-Bonferroni correction. **(C)** Viability, relative to basophils incubated without anti-IgE or disulfiram, as determined by MTS assay, was not reduced by the treatments (n=4). **(D)** Disulfiram did not significantly (ns; determined using a paired Student’s t-test) induce histamine release when incubated with basophils for 45 min at 37°C alone (in the absence of anti-IgE; n=4). Results are expressed as means ± SEM for the indicated number of independent experiments using different basophil donors.

Since the liberation of intracellular calcium is an essential step for basophils to produce and release pro-allergic and other inflammatory mediators, we hypothesized that agents which activate SERCA could potentially inhibit IgE-mediated basophil activation. We therefore used disulfiram which, in addition to its well-known effects as an inhibitor of aldehyde dehydrogenase, also reversibly stimulates SERCA. We observed that disulfiram strikingly and significantly inhibited IgE-dependent basophil histamine release (**Figure 2A** **, B**). Disulfiram did not affect basophil cell viability (**Figure 2C**) and did not markedly induce histamine release from basophils by itself (**Figure 2D**).

## 4 Discussion

Our findings have identified an inverse association between the ability of human basophils to respond to IgE-dependent stimulation and the expressions of SERCA, particularly the SERCA2 isoform. These observations highlight a potential further tier of control of basophil releasability at the level of intracellular calcium mobilization, alongside other known key upstream signals such as SYK and the inhibitory phosphatase SHIP1. In the histamine-releasing rat mast cell line RBL-2H3, Dráberová et al. (36) reported that the non-T cell activation linker (NTAL) regulates store-operated Ca^2+^ channels in FcεRI signalling. However, it is currently not known whether the regulation mediator release to IgE-dependent triggers by the above signals are linked in human basophils.

It has long been known that the SERCA inhibitor, thapsigargin, activates human basophils (25) and potentiates degranulation upon co-stimulation with basophil priming cytokines, such as IL-3 (32), by depleting Ca^2+^ stores. Our results also broadly confirm these earlier findings (**Supplementary Figure 1**). The resulting increases in cytosolic free Ca^2+^ ions open calcium release–activated calcium (CRAC) channels, resulting in a substantial influx of extracellular calcium ions into basophils. Without the influx of extracellular calcium, intermediary signalling is considerably abrogated and mediator secretion from basophils does not occur (12). Our investigations confirm that blocking SERCA function by thapsigargin leads to a slow leakage of intracellular calcium in human basophils which, in the presence of extracellular calcium, results in substantial calcium influx which is comparable to stimulation with the calcium ionophore A23187 (**Supplementary Figure 2**).

The pharmacological activation of SERCA by disulfiram led to a significant inhibition of anti-IgE-stimulated basophils (**Figure 2**), further indicating a role for SERCA in IgE-mediated basophil activation. However, these findings need to be interpreted with caution, since disulfiram is not only a SERCA-specific activator but inhibits acetaldehyde dehydrogenase, inositol 1,4,5-trisphosphate 5-phosphatase (37) as well as vacuolar-type ATPase (V-ATPase) (38). In regards to V-ATPase, Pejler et al. (39) showed that the V-ATPase inhibitor bafilomycin A inhibited IgE-dependent beta-hexosaminidase release from bone marrow-derived mouse mast cells. However, these inhibitory effects were moderate (<50% inhibition) and observed only after long (24h) preincubations with bafilomycin A1. It therefore remains questionable whether the potential inhibitory effects of disulfiram on V-ATPase in basophils is relevant considering the short preincubations (15 min) used in the present study where IgE-dependent histamine release was inhibited within previously published concentrations of the drug required for SERCA stimulation (40).

At the level of SERCA protein expression, the inverse association between constitutive SERCA2 and IgE-induced basophil degranulation clearly underlines a potential role for SERCA2 in determining, at least in part, the magnitude of basophil responses. From our exploratory investigation, this appears to be the case for constitutive IgE-mediated releasability in freshly isolated human basophils but it is not presently clear whether SERCA plays a role in governing basophil releasability in basophils which have been primed by IL-3 or other cytokines (e.g. nerve growth factors, IL-33 etc.) which enhance IgE-dependent mediator release. Importantly, basophil releasability to other stimuli, such as the bacterial peptide fMLP, does not correlate with IgE-mediated histamine release, although the initial Ca^2+^ response (caused by a rise in intracellular free calcium from intracellular stores) was reported to probably arise from the same internal source of the ion (41). This suggests that fMLP-induced basophil degranulation should also be dependent on intitial calcium responses and downregulated by SERCA. However, MacGlashan and Botana previously reported that fMLP-induced histamine release does not correlate with calcium responses (41) and, according to previous reports (32) as well as our own data, are not potentiated by thapsigargin. This suggests a differential dependency on intracellular calcium mobilization and subsequent SERCA input regarding basophil mediator secretion caused by different secretagogues, a point which still requires further clarification.

To the best of our knowledge, this is the first study to implicate a role for SERCA in regulating the function of allergic effector cells. A possible role for diminished SERCA expressions in the context of allergic inflammation is currently not well understood, where studies to date have focussed only on its role in airway smooth muscle cells regarding asthma. Here, Mahn et al. (24) reported that SERCA2 deficiency contributes to a hyperproliferative airway smooth muscle phenotype in asthma and is associated with moderate-severe asthma. These findings, however, were disputed by Sweeney et al. (42) who failed to observe differences in SERCA2 mRNA or protein expressions in airway smooth muscle cells between asthmatic patients and controls. Conversely, Qaisar et al. (43) also reported reduced SERCA expressions in asthma and, in a guinea pig model of asthma, reduced SERCA2b expression in airway smooth muscle cells was recently reported to correlate with intrinsic airway baseline tone (44). The proinflammatory cytokines, TNFα and IL-13, which are heavily implicated in asthma severity, were shown to decrease SERCA2 expressions in human airway smooth muscle cells (23).

SERCA proteins are expressed in at least seven different isoforms (SERCA1a/1b, SERCA 2a/2b, and SERCA 3a/3b/3c), of which only SERCA2b and SERCA3 isoforms are expressed in non-muscle cells (reviewed in (45). We primarily focussed on SERCA2 since this isoform was implicated in asthma in previous reports. However, our preliminary data suggest that human basophils may also constitutively express SERCA3 isoforms (**Supplementary Figure 3**). In contrast, in our preliminary investigations, where we focussed only on constitutive expressions in low or non-responder basophils to IgE-dependent stimulation, we did not observe SERCA1 expression in basophils. Our observations are, in part, supported by previous findings regarding RNA-seq expressions of SERCA2 and 3 (46, 47), which are relatively highly expressed in basophils in comparison to other immune cells (47). However, a gene expression dataset published by Uhlen et al. failed to detect SERCA2 expressions in various granulocytes (including basophils) in contrast to relatively high granulocyte expressions of SERCA3 (48). It is unclear whether the above disparities of SERCA2 gene expressions are related to our observations regarding differential SERCA2 protein expressions with respect to IgE-mediated basophil activation. However, given that human basophils also express SERCA3, further studies are clearly required regarding the possible functional consequences of the differential expressions of various SERCA isoforms in basophils.

Our study was limited to focussing on basophils isolated from buffy coat blood obtained from healthy donors. Further studies are clearly required to determine whether the negative association between SERCA expressions and IgE-mediated basophil releasability has clinical implications, especially regarding allergic diseases. From our exploratory study, there is a clear inverse association between SERCA expression and the ability of human basophils to respond to IgE-dependent stimulation. It remains to be clarified whether SERCA may serve as a new target for therapeutic regulation of basophil responses and is implicated in the functional regulation of other human allergic effector cell types.

## Data availability statement

The raw data supporting the conclusions of this article will be made available by the authors, without undue reservation.

## Ethics statement

The studies involving human participants were reviewed and approved by National Health Service (NHS) Research Ethics Committee (reference number 07/Q1206/3). Written informed consent for participation was not required for this study in accordance with the national legislation and the institutional requirements

## Author contributions

BG conceived the study, conducted most of the experiments, analysed and interpreted the data, and wrote the first draft of the manuscript. AH contributed to the experiments shown in **Supplementary Figures 1 and 3**, with the assistance of VS. All authors contributed to the article and approved the submitted version.
