# Supplementary material for: IgE-dependent human basophil responses are inversely associated with the sarcoplasmic reticulum Ca2+-ATPase (SERCA)
Source: Front Immunol. 2023 Jan 6;13:1052290. doi: 10.3389/fimmu.2022.1052290 (PMC9846818; doi:10.3389/fimmu.2022.1052290)
Supplement: Supplementary file 1 [file DataSheet_1.pdf]

Supplementary Figures

IgE-Dependent Human Basophil Responses are Inversely Associated with the Sarcoplasmic Reticulum Ca2+-ATPase (SERCA)

Anette T. Hansen Selnø[1], Vadim V. Sumbayev[1], Bernhard F. Gibbs[1,2*]

[1]School of Pharmacy, University of Kent, Chatham Maritime, UK
[2]Department of Human Medicine, University of Oldenburg, Oldenburg, Germany

*** Correspondence:**
Bernhard F. Gibbs
bernhard.gibbs@uni-oldenburg.de

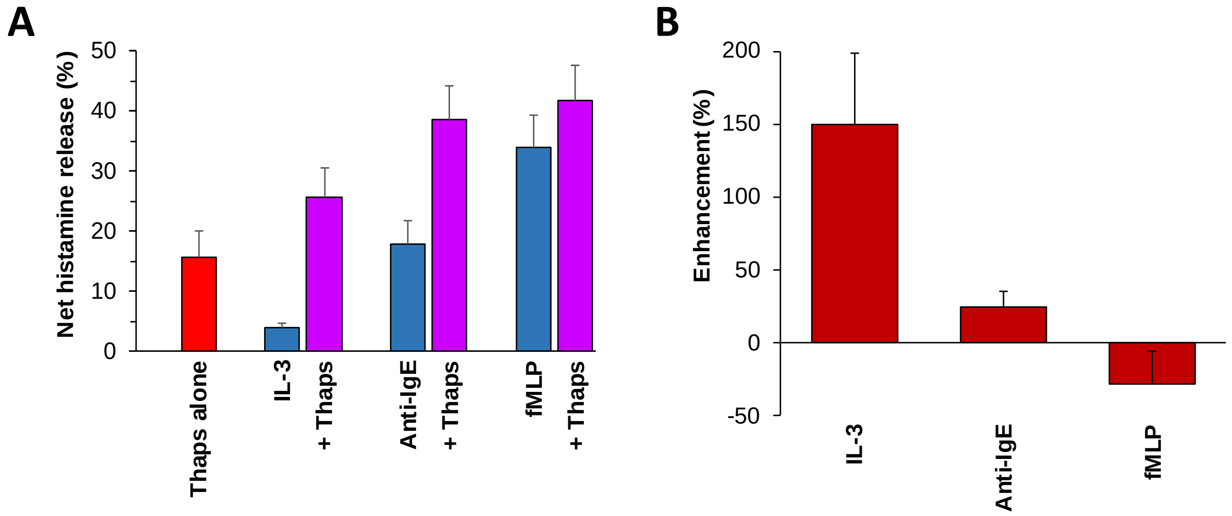

Suppl. fig. 1. Thapsigargin induces histamine release from human basophils and substantially enhances IL-3-mediated - but not anti-IgE- or fMLP-induced - basophil activation
Prewarmed cells were first incubated for 15 min with or without thapsigargin (1 µM) before reacting with or without either anti-IgE (1µg/ml), IL-3 (1 ng/ml), or fMLP (100 nM) (or buffer controls) for a further 30 min. Histamine releases were then determined as described in the Materials and Methods. Results are shown as means ± SEM for n=3 separate experiments (using different basophil donors).

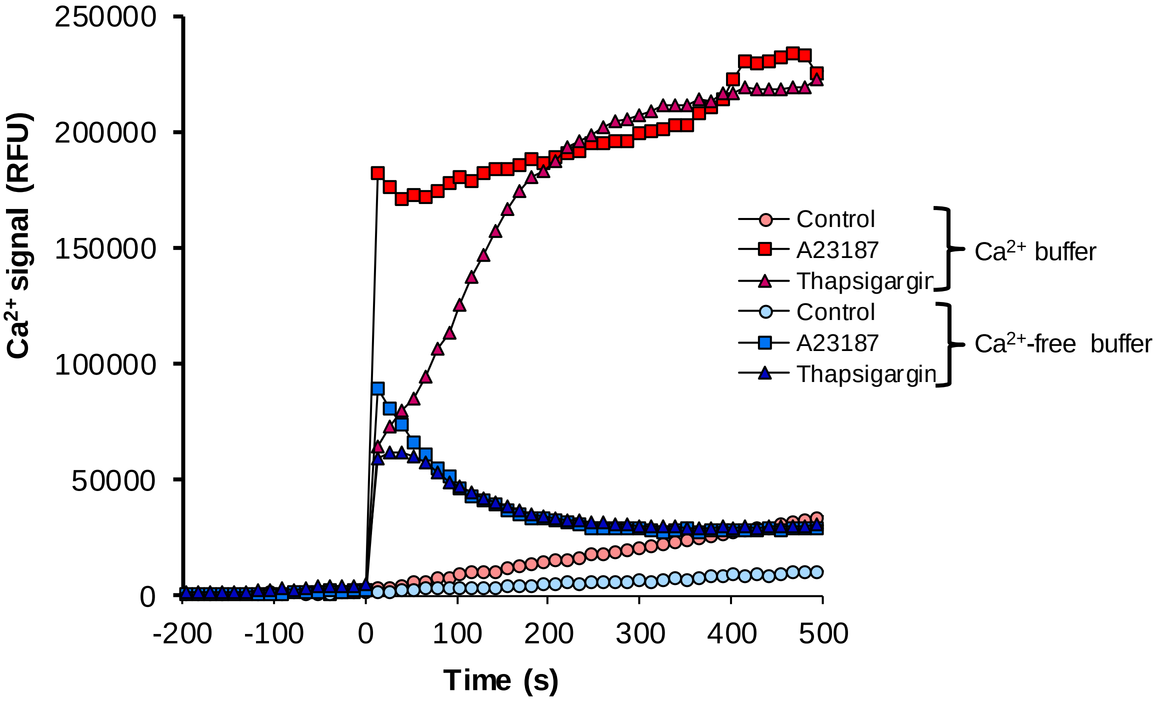

Suppl. fig. 2. Intracellular calcium mobilization in human basophils induced by A23187 and thapsigargin in either the presence or absence of calcium-containing HEPES Tyrode's solution.

Briefly, purified human basophils were loaded with fluo-3 AM (2 µM) for 20 min and, following 3x wash steps resuspended in HEPES-buffered Tyrode's solution, either with or without 1mM $CaCl_2$, and aliquoted into a 96-well culture plate. After a prewarming period for 15 min at 37°C cells were stimulated with or without either A23187 (5 µM; C7522, Sigma-Aldrich, St. Louis, USA) or thapsigargin (1 µM; T9033, Sigma-Aldrich, St. Louis, USA). Calcium mobilization measurements were assayed using a VICTOR3 Multilabel Counter (Perkin Elmer, Waltham, Massachusetts, USA), at an excitation and emission of 506 nm and 526 nm, respectively. Baseline calcium leakage was measured for several minutes prior to cell stimulation and signal intensities shown as relative fluorescence units (RFU). Results were normalized by subtracting the baseline level for the unstimulated control at the first reading for cell incubated wither with or without extracellular calcium.

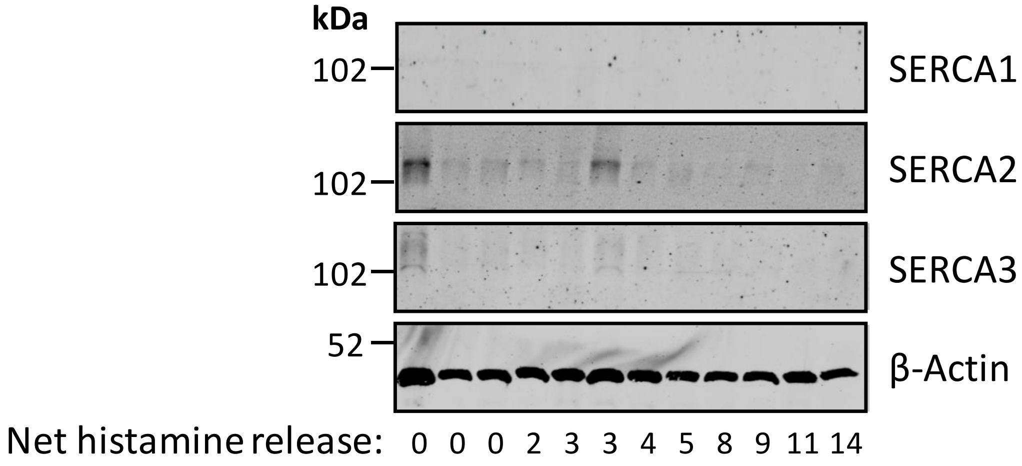

Suppl. fig. 3. Differential expressions of SERCA isoforms in purified human basophils from non-releaser and low-releaser donors to anti-IgE stimulation.

The Western blot shows results for n=12 different donors. Histamine releases and SDS-PAGE were performed as described in the Materials and Methods section. Western blotting was

performed using primary antibodies against various SERCA isoforms, all from Abcam (Cambridge, UK), directed against either human SERCA1 (ab2819, mouse monoclonal), human SERCA2 (ab137020, rabbit monoclonal) or human SERCA3 (ab154259, rabbit polyclonal). The β-actin antibody (mouse monoclonal, A5216) was purchased from Sigma-Aldrich, St. Louis, USA. In contrast to Western blots shown in Fig. 1 (of the main manuscript), which were visualized using HRP-conjugated antibodies and chemiluminescence, the results shown above were visualized using a Li-Cor Odyssey fluorescence imaging system (Lincoln, Nebraska USA). Here, anti-mouse or anti-rabbit secondary antibodies conjugated with fluorescent dyes were employed and proteins were visualized using a Li-Cor Odyssey imager. Please note that we also employed a different SERCA2 primary antibody for the above Western blot in order to confirm the results regarding SERCA2 expressions in basophils shown in Fig. 1.

Donor	SERCA2/β-Actin	Spontaneous histamine release	Net IgE-mediated histamine release
1	1.0640	5.31	9.73
2	1.6958	8.57	18.57
3	0.7151	3.23	24.19
4	0.2973	2.63	34.21
5	0.5725	0.83	25.62
6	0.3952	12.84	33.45
7	0.2418	7.02	48.25
8	0.9663	4.82	15.66
9	0.9946	23.96	22.92
10	0.8731	5.66	16.98
11	1.0000	2.51	4.52
12	1.0134	2.55	8.73
13	0.6263	3.95	21.16
14	0.5719	2.78	30.56
15	0.4132	1.69	35.93
16	0.7853	5.19	31.11
17	0.7414	8.87	26.32
18	0.7101	4.85	14.56
19	1.1861	21.67	6.7

Suppl. table 1. Summary of SERCA2 levels (determined by densitometric analysis of Western blots; as described in the Materials and Methods), spontaneous histamine release and net IgE-dependent histamine secretion (which was calculated from the relative percentage histamine release corrected for respective spontaneous secretion) for all 19 samples shown in Fig.1.
